# Evaluating the effects of vitamin D Level on airway obstruction in two asthma endotypes in humans and in two mouse models with different intake of vitamin D during early-life

**DOI:** 10.3389/fimmu.2023.1107031

**Published:** 2023-01-30

**Authors:** Yan Zhou, Yali Qiu, Wuping Bao, Lei Han, Yishu Xue, Yingying Zhang, Xue Tian, Qiang Fu, Chengjian Lv, Dongning Yin, Min Zhang

**Affiliations:** ^1^ Department of Respiratory and Critical Care Medicine, Shanghai General Hospital, Shanghai Jiao Tong University School of Medicine, Shanghai, China; ^2^ Department of Respiratory and Critical Care Medicine, Shanghai General Hospital of Nanjing Medical University, Shanghai, China

**Keywords:** vitamin D, airway inflammation, airway resistance, asthma endotypes, T2-low asthma

## Abstract

**Introduction:**

Asthma is primarily divided into two categories: type 2 (T2-high) and non-type 2 (T2-low). A relationship between asthma severity and vitamin D deficiency has been identified, but its impact on each asthma endotype remains unknown.

**Methods:**

We clinically examined the influence of vitamin D on patients with T2-high (n = 60) or T2-low asthma (n = 36) compared with controls (n = 40). Serum 25(OH)D levels, inflammatory cytokines and spirometry were measured. Mouse models were then used to further analyze the effects of vitamin D on both asthmatic endotypes. BALB/c mice were fed with vitamin D-deficient (LVD), -sufficient (NVD), or -supplemented diets (HVD) throughout lactation and offspring followed the same diet after weaning. Offspring were sensitized/challenged with ovalbumin (OVA) to establish “T2-high” asthma or OVA combined with ozone exposure (OVA + ozone) to induce “T2-low” asthma. Spirometry and serum, bronchoalveolar lavage fluid (BALF), and lung tissues were analyzed.

**Results:**

Serum 25(OH)D levels were decreased in asthmatic patients compared with controls. Patients with vitamin D deficiency (Lo) had varying degrees of elevation of the pro-inflammatory cytokines IL-5, IL-6, and IL-17A, decreased expression of the anti-inflammatory cytokine IL-10, and altered forced expiratory volume in the first second as a percentage of predicted value (FEV_1_%pred) in both asthmatic endotypes. Vitamin D status had a stronger correlation with FEV_1_%pred in T2-low asthma than T2-high asthma, and 25(OH)D level was only positively linked to maximal mid-expiratory flow as a percentage of predicted value (MMEF%pred) in the T2-low group. Inflammation, hyperresponsiveness, and airway resistance (R_L_) was increased in both asthma models compared with controls while vitamin D deficiency further increased airway inflammation and airway obstruction. These findings were particularly prominent in T2-low asthma.

**Discussion:**

The potential function and mechanisms of vitamin D and both asthma endotypes should be studied individually, and further analysis of the potential signaling pathways involved with vitamin D on T2-low asthma is warranted.

## 1 Introduction

Asthma is a heterogeneous chronic airway inflammatory disease that is characterized by two main inflammatory endotypes: Type 2 (T2)-high and T2-low/non-T2 (1–3). T2-high asthma is characterized by a lymphocyte T helper 2 (Th2)-and innate lymphoid cell 2 (ILC2) -driven immune-inflammatory response (4). In contrast, the T2-low endotype is characterized by neutrophil-dominated inflammation that is consistent with either a type 1 immune response, type 3 inflammation mediated by Th17 cytokines, systemic inflammation associated with IL-6 release and obesity, or the lack of an inflammatory process known as the paucigranulocytic endotype (5–7). T2-low asthma is a distinct, adult-onset, severe, more steroid-refractory subtype that is associated with comorbidities such as obesity and gastroesophageal reflux (8). Common maintenance treatments for asthma, such as inhaled corticosteroids (ICS) or antileukotrienes, and new biotherapies against innate immunity-driven neutrophilic inflammation perform poorly against T2-low asthma (9–11). Asthma pathogenesis may involve vitamin D deficiency (12, 13), meriting an evaluation of the effects of vitamin D on both asthma endotypes, especially T2-low.

The most active vitamin D metabolite, 1,25-dihydroxyvitamin D_3_ (1,25(OH)_2_D_3_) plays an important role in the innate immune response (14, 15). Groot et al. (16) found that vitamin D supplementation significantly reduced eosinophilic airway inflammation in asthmatics but did not affect induced sputum neutrophil count. Other studies have reported that vitamin D has an immunosuppressive effect on Th17 cells (17–19). Nanzer et al. (17) recruited eighteen patients with steroid-resistant (SR) asthma and 10 patients with steroid-sensitive (SS) asthma were assessed with a mean age of 54 (SR) and 50 (SS) years. Human Peripheral blood mononuclear cells (PBMCs) were isolated, and stimulated in culture with or without 10^-7^mol/L dexamethasone and 1,25(OH)_2_D3 *in vitro*. Nanzer et al. showed that the expression of IL-17A in the peripheral blood of asthmatics could not be reduced with glucocorticoids but could be curbed with 1,25(OH)_2_D_3_ supplementation. However, clinical trial results to this end are conflicting, suggesting that vitamin D supplementation is unlikely to reduce the risk of atopic disease. Manousaki D et al (20) found that four single-nucleotide polymorphisms (SNPs) strongly associated with 25-hydroxyvitamin D [25(OH)D] levels in 33,996 individuals, and conducted Mendelian randomization (MR) studies to estimate the effect of lowered 25(OH)D on the risk of asthma, atopic dermatitis, childhood onset asthma, and elevated IgE level and tested MR assumptions in sensitivity analyses. They found no evidence that genetically determined reduction in 25(OH)D levels conferred an increased risk of asthma, atopic dermatitis, or elevated total serum IgE. Yepes-Nunez JJ et al (21) searched three databases through January 30, 2016, including nonrandomized studies (NRS) and randomized (RCT). Among the 1932 articles identified, four NRS and one RCT were eligible. Their studies suggests that vitamin D supplementation for pregnant women, breastfeeding women, and infants may not decrease the risk of developing allergic diseases such as atopic dermatitis. Possible reasons for this include a poor understanding of which patients would benefit the most from supplementation and when vitamin D supplementation would be most effective. Current stratified approaches that treat asthma based on phenotype have shortcomings, requiring the definition of unbiased multidimensional endotypes to account for the complexities of this disease. Further studies are needed to confirm the effects of vitamin D on specific asthma endotypes.

Mechanistic studies in human and animal models have shown that vitamin D is involved in immune cell function and fetal lung development and maturation (22, 23). Vitamin D deficiency early in life may increase the risk of asthma as the child grows. We therefore evaluated the effects of vitamin D status on both asthma endotypes using infant mouse models.

Our previous work showed that female BALB/c were provided with vitamin D-deficient, -sufficient or -supplemented diets throughout lactation and their pups followed the same diet after weaning. Offspring were then sensitized and challenged with OVA, vitamin D supplementation can reduce allergic airway inflammation and hyperresponsiveness (AHR) in OVA-mediated models (24). We therefore sought to evaluate the effects of vitamin D on the T2-high and T2-low asthma endotypes in humans and a mouse model.

## 2 Materials and methods

### 2.1 Study design and participants

**Figure 1:**
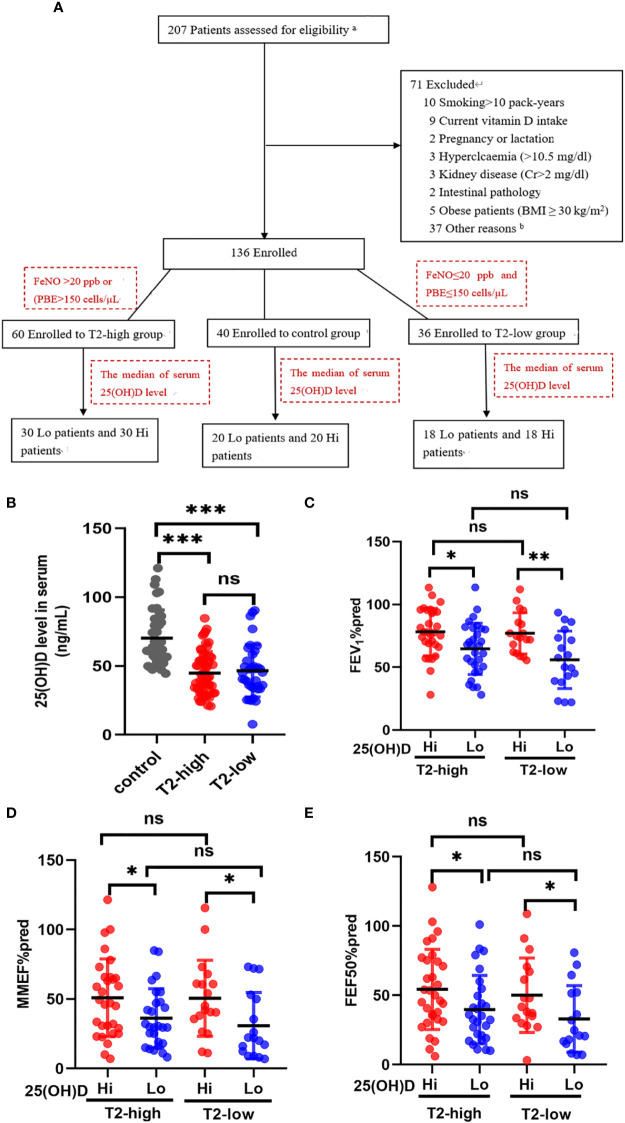
The effects of serum 25(OH)D level on FEV_1_%pred and small-airway variables in both asthma groups. **(A)** The experiment design. **(B)** The serum levels of 25 (OH)D were significantly lower in both asthma endotypes compared with controls. **(C–E)** The effects of different serum 25(OH)D levels on FEV_1_%pred, MMEF%pred, and FEF50%pred in the T2-low and T2-high groups. * P < 0.05, ** P < 0.01, and *** P < 0.001. ns, non-significant. a Patients selected from list of patients (hospitalised in department of Respiratory and Critical Care Medicine in 2020–2022). b Not possible to contact patients. PBE, blood eosinophil count; Lo, vitamin D-deficient group; Hi, vitamin D-sufficient. FEV1%pred, forced expiratory volume in the first second as a percentage of predicted value; FEF50, forced expiratory flow at 50% of FVC; MMEF, mean mid expiratory flow, average flow from 25–75% FVC.

Our trial design was a cross-sectional observational study conducted between August 2020 to July 2022 from two Shanghai General Hospital facilities (Shanghai, China). The aim was to investigate the relationship between different serum 25(OH)D levels and airway obstruction in patients with asthma. Of the 207 possible participants were identified. Demographic characteristics (age, gender, body mass index and smoking history), symptoms, serum 25-hydroxyvitamin D (25(OH)D) levels, blood routine examination, serum inflammatory cytokines, fractional exhaled nitric oxide (FeNO), pulmonary function, and treatments were extracted from the electronic medical record during a follow-up visit. The detailed history included the most common comorbidities. After checking all the inclusion and exclusion criteria, 136 patients agreed to participate in the trial (**Figure 1A**). 60 patients with T2-high asthma and 36 patients with T2-low asthma were assessed with a mean age of 52 years, 25 (42%) were male (T2-high), and mean age of 52 years, 14 (39%) were male (T2-low). Fourty control subjects were assessed for comparison [mean age of 59 years, and 19 (47%) were male]. Each of the above groups was divided into vitamin D-deficient (Lo) or vitamin D-sufficient (Hi) according to the median of serum 25(OH)D level. The main baseline characteristics of patients are summarised in **Table 1**.

**Table 1:** Baseline asthma patient characteristics.

Characteristic	T2-high (n=60)Hi (30) Lo (30)	T2-low (n=36)Hi (18) Lo (18)	Control (n=40)Hi (n=20) Lo (20)	*p*-value	
Age, year	56.03 ± 13.45 53.13 ± 13.63	52.41 ± 15.40 58.18 ± 13.03	61.06 ± 11.67 58.24 ± 11.66	NS	
Male, n (%)	14 (46.67) 11 (36.67)	6 (33.33) 8 (44.44)	7 (46.67) 12 (48.0)	NS	
BMI, kg/m^2^	24.41 ± 3.77 24.38 ± 3.42	24.00 ± 3.06 23.97 ± 3.71	23.09 ± 4.16 24.40 ± 2.92	NS	
Current smokers	2 (6.67%) 1 (3.33%)	1 (5.56%) 1 (5.56%)	1 (6.67%)1 (4.0%)	NS	
Former smokers	2 (6.67%) 2 (6.67%)	2 (11.11%) 2 (11.11%)	2 (13.33%) 3 (12%)	NS	
Duration of disease (years)	5.93 ± 1.97 8.17 ± 2.38	7.17 ± 2.32 5.98 ± 1.71	--	NS	
Exacerbations, n	2.70 ± 0.49 2.72 ± 0.64	2.78 ± 0.56 2.67 ± 0.59	--		
ICS dose, n (%)			--		
Low	6 (20%) 6 (20%)	2 (11.11%) 1 (5.56%)	--	NS	
Medium	10 (33.3%) 9 (30%)	7 (38.89%) 5 (27.78%)	--	NS	
High	14 (46.67%) 15 (50%)	9 (50%) 12 (66.67%)	--	NS	
Oral corticosteroid, n (%)	5 (16.67%) 10 (33.3%)	7 (38.89%) 7 (38.89%)	--	NS	
Oral corticosteroid dose (mg)	5.23 ± 1.73 10.55 ± 3.73^#^	7.36 ± 2.58 13.51 ± 4.25^#^	--	<0.001	
25 (OH)D (nmol/L)	56.49 ± 1.92 31.55 ± 1.05^#^	58.00 ± 3.28 32.27 ± 2.06^#^	82.53 ± 3.54^*^ 53.26 ± 1.11^*^	<0.001	
ACT scores	17.68 ± 2.51 15.43 ± 3.19^#^	17.16 ± 2.52 14.21 ± 3.66^#^	--	<0.001	
Calcium (mg/dL)	2.28 ± 0.11 2.24 ± 0.67	2.30 ± 0.79 2.23 ± 0.99	2.22 ± 0.11 2.23 ± 0.99	NS	
FVC%	83.55 ± 17.37 75.58 ± 16.61	80.38 ± 15.81 68.75 ± 18.70^#^	93.31 ± 15.58^*^ 94.09 ± 14.20^*^	<0.001	
PEF%	72.37 ± 19.62 67.63 ± 30.35	74.86 ± 20.38 58.86 ± 29.81^#^	90.20 ± 21.53^*^ 91.06 ± 17.43^*^	<0.001	
FEV1/FVC%	76.51 ± 8.56 63.82 ± 17.66	75.77 ± 10.71 69.53 ± 13.14	82.05 ± 7.91^*^ 83.55 ± 6.15^*^	<0.001	
Eosinophilic count (cells/μL)	385.9 ± 358.37 647.1 ± 645.33	112.8 ± 93.92^Ϯ^ 108.3 ± 107.94^Ϯ^	132.8 ± 99.52^*^ 134.5 ± 95.06^*^	<0.001	

Data are presented as a mean ± standard deviation (SD), unless otherwise indicated. BMI, body mass index; Hi, vitamin D-sufficiency; Lo, vitamin D deficiency; NS, non-significant; ICS dose: inhaled corticosteroids; according to the criteria defined by the global initiative for asthma. Available from: www.ginasthma.com FEV1, forced expiratory volume in the first second; FVC, forced vital capacity; PEF, Peak Expiratory Flow; ACT, asthma control test (ACT) scores. ICS dose: according to the criteria defined by the global initiative for asthma. Available from: www.ginasthma.com. NS, no significant. *There were statistical differences between the control group and the asthma group. ^#^There were significant differences between Hi group and Lo group in athmatic patients. †There were statistically significant differences between T2-High and T2-Low groups in asthmatic patients.

At least three spirometry tests were performed per subject, with the highest values used in our correlation analysis. The diagnosis of asthma was based on the Global Initiative for Asthma (GINA) 2020 guidelines (26). Participants were classified as T2-high (FeNO>20 ppb or blood eosinophil count (PBE)>150 cells/µL) or T2-low (FeNO ≤ 20 ppb and PBE ≤ 150 cells/µL) at the time of enrollment and at the time of each exacerbation (27).

This study excluded pregnant women, patients who were actively breast feeding, patients with a co-morbidity other than asthma that might affect their serum 25(OH)D levels, and patients receiving nutritional supplements that could influence their serum 25(OH)D levels. Human studies were reviewed and approved by the Ethics Committee of Shanghai General Hospital (No. 2018KY186) and registered at the Chinese Clinical Trial Registry (chictr.org.cn, no. ChiCTR2000029065). All participants provided informed consent at the time of recruitment and were followed at one of two centers of Shanghai General Hospital (Shanghai, China).

### 2.2 25(OH)D measurement

Serum 25(OH)D, a widely used indicator of vitamin D status, was detected using radioimmunoassay (RIA) kits (DiaSorin, Stillwater, MN, USA) following the manufacturer’s instructions. The median of serum 25(OH)D level was used to classify asthmatic patients as either vitamin D-deficient (Lo) or vitamin D-sufficient (Hi) (28). Mouse serum 25(OH)D levels were measured using an enzyme linked immunosorbent assay (ELISA) following the manufacturer’s instructions (R&D Systems China Co. Ltd., Shanghai, China).

### 2.3 The mice were provided different concentration of vitamin D diets

Pregnant BALB/c mice (14 days of pregnancy) were purchased from SLAC Laboratory Animal Co. Ltd. (Shanghai, China) and individually housed under controlled temperature (23 ± 2°C), humidity (50 ± 10%), and light/dark cycle (12 h/12 h) conditions in a pathogen-free room. During this time, all the pregnant mice were provided standard food containing 1000 IU vitamin D/Kg. Once the offspring were born, the maternal mice were separately fed with either vitamin D-deficient (LVD) (0 IU vitamin D/kg) (10), vitamin D-sufficient (NVD) (1000 IU vitamin D/kg) (25, 29, 30), or vitamin D-supplemented (HVD) diets (2280 IU vitamin D/kg) (31) (Xietong Laboratories, Jiangsu, China). As with the NVD and HVD diets, the LVD diets were supplemented with vitamins A, E, and K and 1.2% calcium (29, 32). The offspring had the same diet as their mother after weaning, and food intake was measured every other day and were main experimental subjects of our following study. The experimental diet composition (Research Diets, Inc., New Brunswick, NJ, USA) is provided in **Supplementary Table 1** of Supplementary information. The numbers of female and male offspring studied in this research are show in **Supplementary Table 2** of Supplementary information.

### 2.4 Allergic airway inflammation induction and ozone exposure

Mice in the three diet groups were randomly divided into three additional groups (n=8 per group): controls, OVA and OVA + ozone. The mice in the OVA and OVA + ozone groups were sensitized intraperitoneally (IP) with 20 µg OVA (Grade V, Sigma-Aldrich) diluted in 0.2 mL of Dulbecco’s phosphate buffered saline (PBS) and 2 mg aluminum hydroxide (Sigma-Aldrich) in a total volume of 20 mL on days 0, 7, and 14, and challenged via aerosol nebulization with 1% OVA for 30 min each day from day 21 to day 25. Mice in the control group were treated with PBS at both timepoints in the same manner as previously described (33). After the OVA challenge, the same number of mice in the OVA + ozone group were exposed to 2.5 ppm ozone for 2 h daily from day 21 to day 25 as previously described (34, 35). The control mice were exposed to room air during this period.

### 2.5 Spirometry using the forced manoeuvres system

Twenty-four hours after the last OVA challenge, the mice were anesthetized, fitted with a tracheal cannula, and attached to a plethysmograph with a pneumotachograph using the eSpira Forced Manoeuvres System for mice (EMMS, Hants, UK) to mimic classical clinical spirometry (36). We measured FEF50 and MMEF as indicators of small-airway function.

### 2.6 Airway responsiveness

The anesthetized mice were then ventilated (MiniVent, Hugo Sachs Electronik, Germany) in a whole-body plethysmograph with a pneumotachograph linked to a differential pressure transducer (EMMS, Hants, UK) (36). Airway resistance (R_L_) was recorded for 3 min at each concentration of inhaled acetylcholine chloride (ACh, Sigma-Aldrich, USA) (4-256 mg/mL, 10 µL each time). R_L_ was expressed as the percentage change from baseline R_L_ (measured following PBS nebulization). The ACh concentration required to increase R_L_ by 100% from baseline was calculated (PC100), and -logPC100 was used as an indicator of AHR.

### 2.7 Bronchoalveolar lavage fluid measurements

The tracheas of the anesthetized mice were accessed to collect bronchoalveolar lavage fluid (BALF) *via* infusion of 0.6 mL PBS three times through a polyethylene (PE-60) tube (37). The BALF recovery rate was above 80% (37). The reclaimed BALF was centrifuged at 3000 r/min for 10 min at 4°C. Supernatants were collected to measure the expression of IL-4, IL-6, IL-10, IL-17A, IL-1β and TNF-α *via* ELISA using kits (Invitrogen, CA, USA) following the manufacturer’s protocol. Cell counts were performed using Wright–Giemsa stained cytospin slides by two independent, blinded investigators.

### 2.8 Histologic and morphometric analysis

The left lungs of the mice were fixed in 4% paraformaldehyde for 24 h, embedded in paraffin, sectioned to expose the maximum surface area of the lung tissue in the plane of the bronchial tree, and stained with hematoxylin-eosin (HE) (38). Peribronchiolar and perivascular area were observed in the HE stained lung slices, and each tissue section was scored on a scale from 0 to 3 (36). Airway inflammatory cell infiltration density (per 100 μm) was calculated in a double-blinded manner by two independent investigators.

### 2.9 Statistical analysis

**Figure 2:**
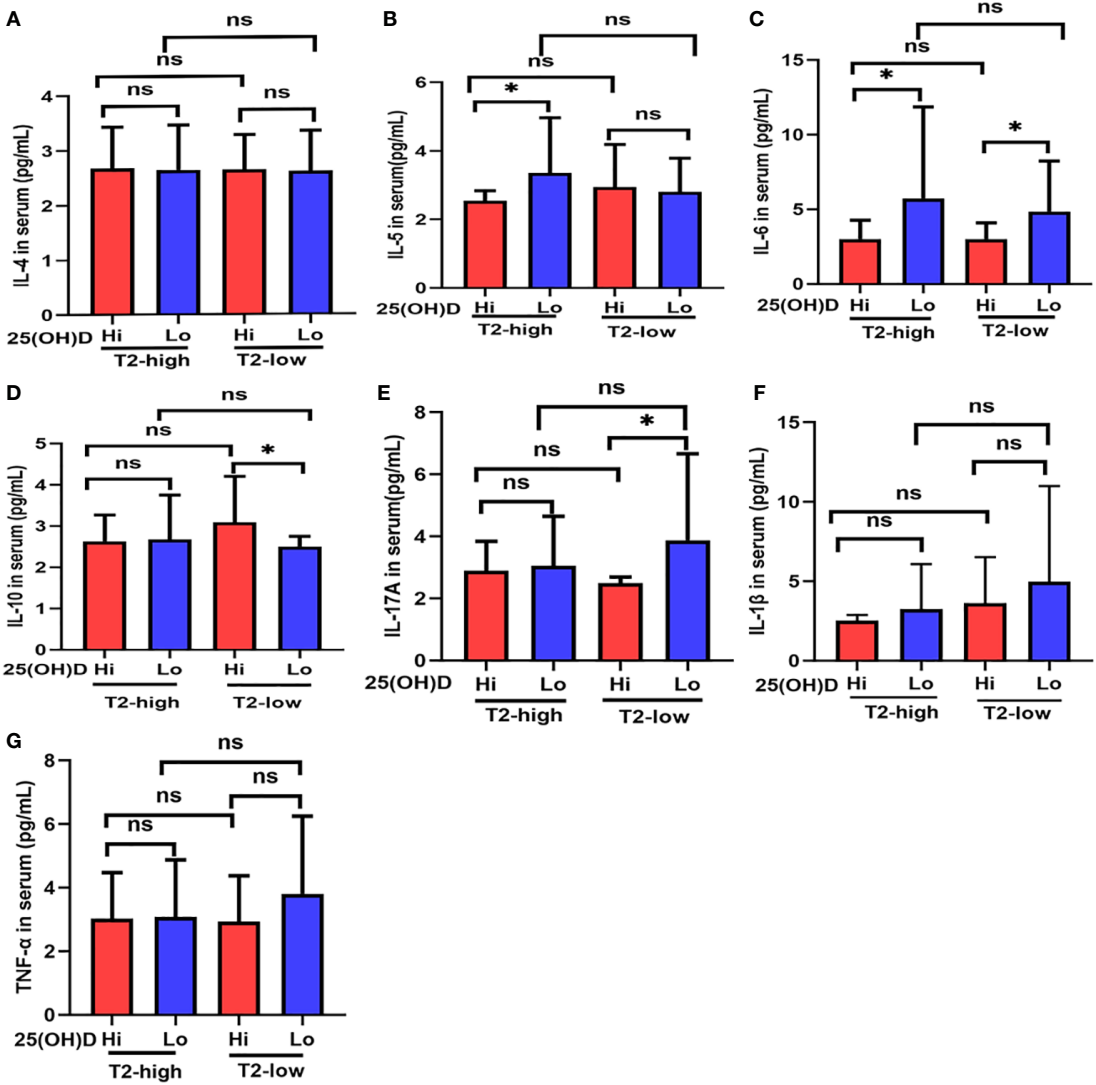
The expression of **(A)** IL-4, **(B)** IL-5, **(C)** IL-6, **(D)** IL-10, **(E)** IL-17A, **(F)** IL-1β, and **(G)** TNF-α at different serum 25(OH)D levels in asthmatic patients. * P < 0.05, ns, non-significant; IL, interleukin; TNF, Tumor Necrosis Factor.

All statistical analyses were performed using SPSS 25.0. Continuous variables were expressed as mean ± standard deviation (SD) or median plus range, and categorical variables were expressed as number (%). One-way analysis of variance (ANOVA) and the Student–Newman–Keuls (S-N-K) *post hoc* test were used to compare multiple groups. Categorical variables were compared using the Chi-square (χ^2^) test. A correlation heatmap as shown in **Figures 2A, B** was visualized using GraphPad Prism 8.0 (La Jolla, CA, USA). *P* < 0.05 was considered statistically significant.

## 3 Results

### 3.1 Demographic characteristics and physical performance

A total of 96 patients with asthma (mean age, 55.9 ± 13.74) were included and categorized into T2-high (n=60), T2-low (n=36), and control (n=40) groups. There were no significant differences between the asthmatic patients and control subjects in age, height, body mass index, ICS dose, numbers of previous exacerbations and smoking history (**Table 1**). The Asthma Control Test (ACT) provides a standardized score for helping healthcare professionals and patients assess whether asthma symptoms are well managed. The average ACT scores of patients with vitamin D deficiency (Lo) were significantly lower than those with vitamin D sufficiency (Hi) in both asthmatic endotypes. There were significant differences between asthmatics and control subjects in FVC%, PEF%, and FEV_1_/FVC%, which were all lower in asthmatics. FVC% and PEF% were lower in the Lo group compared with the Hi group in T2-low patients, but not in T2-high patients. FEV_1_/FVC% were lower in both asthma groups compared with controls, but equivalent between T2-high and T2-low. Eosinophil levels were significantly elevated in the T2-high group compared with the T2-low and control groups, and higher in Lo *vs*. Hi patients in the T2-high group (**Table 1**).

### 3.2 FEV_1_%pred and small-airway variables were decreased in patients with vitamin D deficiency regardless of asthma endotype

Serum 25 (OH)D levels were significantly lower in the T2-high and T2-low groups compared with controls (**Figure 1B**). Patients with Lo had decreased FEV_1_%pred and small-airway variables (MMEF%pred and FEF50%pred) compared with Hi in both asthmatic groups (**Figures 1C–E**). The FEV_1_%pred, MMEF%pred, and FEF50%pred of patients with vitamin D deficiency in the T2-low group were lower those with vitamin D deficiency in the T2-high group, but these relationships were not significant (**Figures 1C–E**).

### 3.3 Serum inflammatory cytokines were elevated in patients with vitamin D deficiency

Serum IL-5 levels in Lo patients were significantly higher than those with Hi in the T2-high group (**Figure 2B**), and IL-17A expression had a similar effect in the T2-low group (**Figure 2E**). IL-6 expression was higher in Lo patients compared with Hi patients in both asthmatic groups (**Figure 2C**). Serum IL-10 expression in Lo patients was less than Hi patients in the T2-low group, but not in the T2-high group (**Figure 2D**). IL-4, IL-1β, and TNF-α expression in the Lo group were higher than Hi patients in both asthmatic groups, but these relationships were not significant (**Figures 2A, F, G**).

### 3.4 Serum 25(OH)D levels correlated with FEV_1_%pred and serum inflammatory cytokines

**Figure 3:**
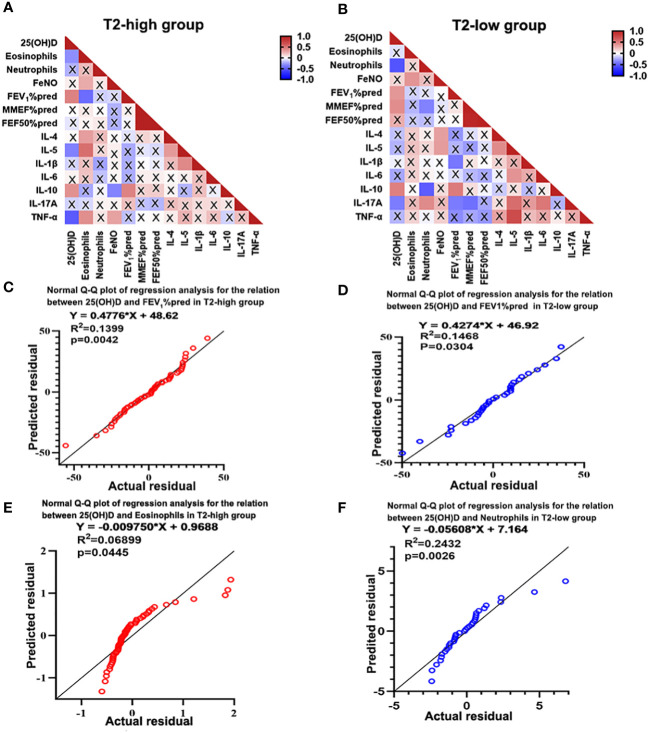
Heatmap of Spearman’s correlation between serum 25(OH)D level and inflammatory cytokines and lung function. **(A)** Serum 25(OH)D level was negatively correlated with eosinophils, IL-5 and TNF-α, and positively correlated with IL-10 and FEV_1_%pred in the T2-high group. **(B)** Serum 25(OH)D level was negatively correlated with neutrophils and IL-17A, and positively correlated with IL-10 and FEV_1_%pred in the T2-low group. **(C, D)** Regression analysis revealed that 25(OH)D may contributed to increased FEV_1_%pred in both asthma groups. **(E, F)** Regression analysis revealed that 25(OH)D may contribute to reduced eosinophils in the T2-high group and decreased neutrophils in the T2-low group. A cross represents no statistical significance. *p*-Values are shown in **Supplementary Tables 3**, **4** of Supplementary information. IL, interleukin; TNF, Tumor Necrosis Factor; FEV1%pred, forced expiratory volume in the first second as a percentage of predicted value.

Correlation was evaluated using Spearman’s rank analysis. Serum 25(OH)D levels were negatively correlated with eosinophil count (r=-0.301, *P*=0.021) and IL-5 (r=-0.354, *P*=0.016) and TNF-α expression (r=-0.503, *P*=0.001) in the T2-high group, and negatively correlated with neutrophil count (r=-0.058, *P*=0.001) and Th17 related cytokine IL-17A expression (r=-0.397, *P*=0.040) in the T2-low group. However, the serum 25(OH)D levels were positively correlated with IL-10 expression (r=0.366, *P*=0.017 in T2-high and r=0.474, *P*=0.008 in T2-low) and FEV_1_%pred (r=0.432, *P*=0.001 in T2-high and r=0.377, *P*=0.033 in T2-low) in the two groups. (**Figure 3A**). However, the serum 25(OH)D status only were positively associated with MMEF%pred (r=0.415, *P*=0.022) in T2-low group (**Figure 3B**). Further regression analysis revealed that 25(OH)D might contribute to elevated FEV_1_%pred (R^2^ = 0.1399, regression coefficient = 0.4776, **Figure 3C** and R^2^ = 0.1468, regression coefficient = 0.4274; **Figure 3D**) in two groups. Meanwhile, regression analysis revealed that 25(OH)D decreased eosinophil count (R^2 ^= 0.0455, regression coefficient=0.00975, **Figure 3E**) in the T2-high group and neutrophil count (R^2 ^= 0.2432, regression coefficient = 0.05608; **Figure 3F**) in the T2-low group. Although the regression coefficients were low, they were statistically significant.

### 3.5 Vitamin D deficiency diets decreased mouse serum 25(OH)D concentration

**Figure 4:**
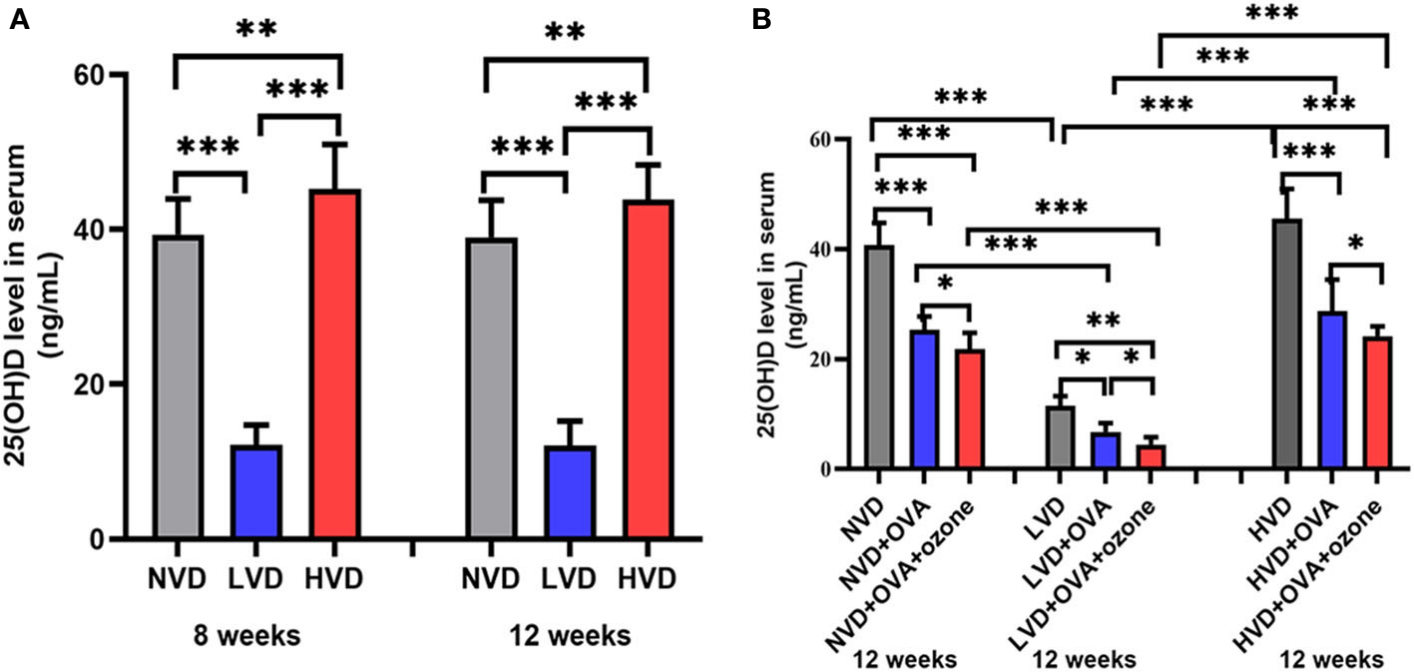
**(A)** Serum 25(OH)D concentration in 8-week and 12-week-old offspring mice with NVD, LVD, or HVD at birth. **(B)** At 12 weeks of age, serum 25(OH)D levels in the OVA groups, OVA + ozone groups and corresponding control groups were measured. Values are mean ± SD (n = 8 per group). *P< 0.05, **P < 0.01, and ***P < 0.001. NVD, vitamin D sufficiency; LVD, vitamin D deficiency; HVD, vitamin D-supplementation.

The serum 25(OH)D levels were 40.63 ± 5.18 ng/mL, 11.60 ± 1.89 ng/mL, 48.14 ± 6.48 ng/mL in offspring fed NVD, LVD and HVD diets, respectively. The serum 25(OH)D concentration was lower in the LVD group than the NVD or HVD groups in 8-week-old and 12-week-old mice. The three groups with different diets did not have significant differences on the serum 25(OH)D concentrations in 8-week-old and 12-week-old mice (**Figure 4A**). Serum levels of 25(OH)D were decreased compared with controls after OVA alone or OVA + ozone co-exposure, and further reduced in the LVD groups than the NVD and HVD groups (**Figure 4B**).

### 3.6 The effects of vitamin D diets on airway resistance, small-airway variables and airway hyperresponsiveness

**Figure 5:**
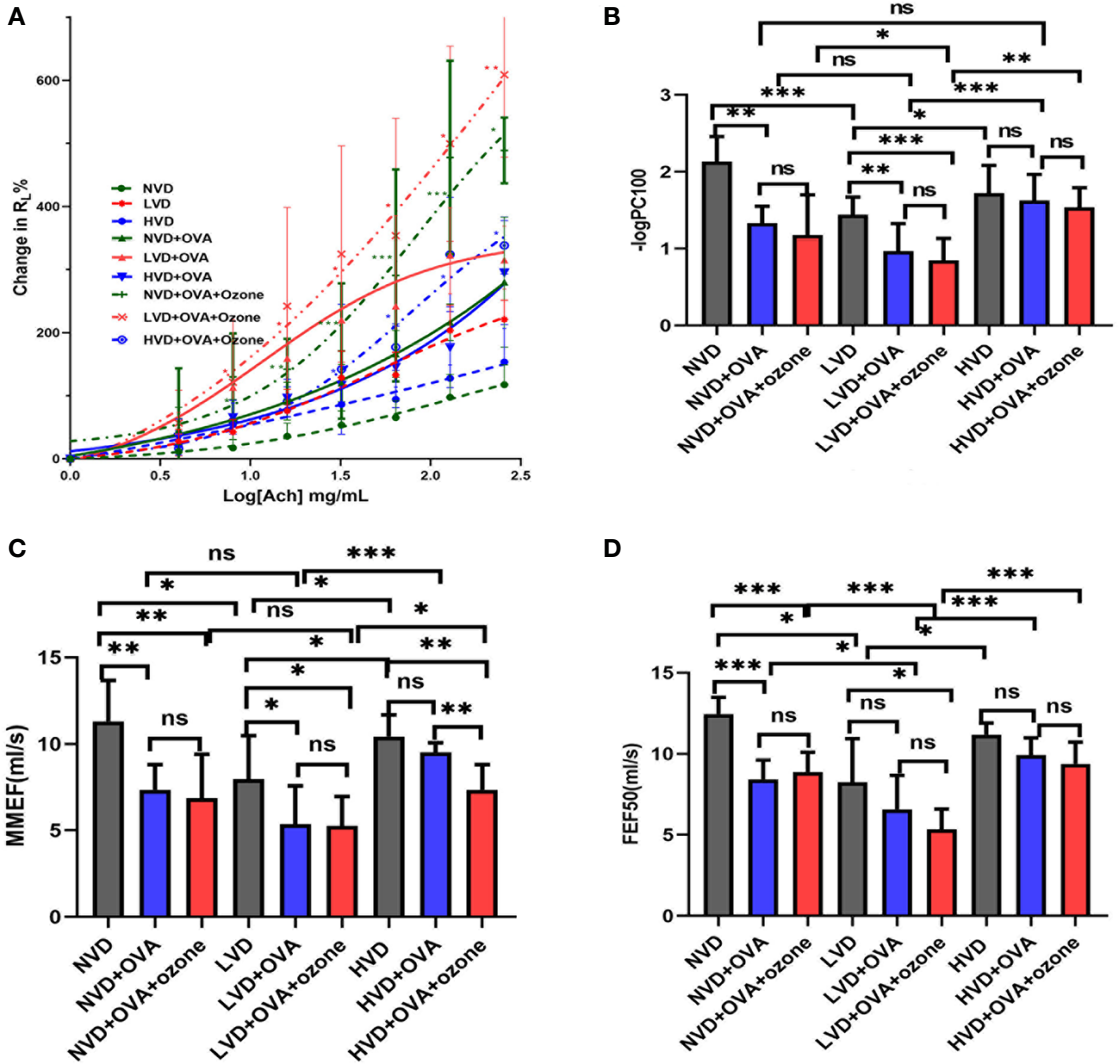
The effects of different vitamin D diets on lung function in mouse asthma models. **(A)** The mean percentage of R_L_ increased with inhaled ACh concentration. The smaller group are shown in **Figure S2** of Supplementary information. **(B)** -logPC100 (ACh concentration increased R_L_ by 100% from baseline) of the nine groups is shown in Panel **(B)** Data are expressed as mean ± SD of eight animals in each group. **(C)** Vitamin D deficiency decreased the MMEF of both mouse models. **(D)** Vitamin D deficiency decreased the FEF50 of both mouse models. * P < 0.05, ** P < 0.01, and *** P < 0.001 compared to the control group shown in different colors. ns, non-significant. ACh, acetylcholine chloride; NVD, vitamin D-sufficient group; NVD + OVA, OVA-sensitized/challenged mice in vitamin D-sufficient group; NVD + OVA + ozone, OVA-sensitized/challenged mice with ozone exposure in vitamin D-sufficient group; LVD, vitamin D-deficient group; LVD + OVA, OVA-sensitized/challenged mice in vitamin D-deficient group; LVD + OVA + ozone, OVA-sensitized/challenged mice with ozone exposure in vitamin D-deficient group; HVD, vitamin D supplemented group; HVD + OVA, OVA sensitized/challenged mice in vitamin D-supplemented group; HVD + OVA + ozone, OVA sensitized/challenged mice with ozone exposure in vitamin D-supplemented group; MMEF, mean mid expiratory flow, average flow from 25–75% FVC; FEF50, forced expiratory flow at 50% of FVC.

There were no obvious differences in baseline R_L_ following buffered PBS nebulization between the nine groups (**Figure 5A**). Mice in the LVD control group had a leftward shift in their R_L_ concentration responsiveness curves compared with the NVD and HVD control groups, while there were no significant differences in the percentage change from baseline R_L_ at different concentrations (**Figure 5A**). After OVA or OVA + ozone exposure, animals in the three different diet groups all had a leftward shift in their R_L_ concentration responsiveness curves, representing increased airway responsiveness compared with control mice on the same diets. The mouse models given LVD diets had stronger airway responsiveness and increased R_L_ at 8 mg/mL, 16 mg/mL, 64 mg/mL, 128 mg/mL, and 256 mg/mL ACh concentrations compared with those fed NVD and HVD diets (**Figure 5A**). declined dramatically after OVA or OVA + ozone exposure in the LVD and NVD groups, indicating AHR in the LVD and NVD groups (**Figure 5B**). The –logPC100 decreased significantly after OVA + ozone exposure in the LVD group compared with NVD and HVD group.

The mice fed LVD diets had lower MMEF and FEF50 compared with those fed NVD and HVD diets. (**Figures 5C, D**). Except for HVD diets after OVA sensitization/challenge, the MMEF further declined in the LVD and NVD groups after OVA or OVA + ozone exposure. The MMEF was lower in the HVD + OVA + ozone group than the HVD + OVA group, but no significant differences were observed between HVD and HVD + OVA groups (**Figure 5C**). The FEF50 further declined in the NVD groups after OVA or OVA + ozone exposure, and the LVD + OVA + ozone group had a lower FEF50 than the LVD control group, but there were no obvious differences between LVD and LVD + OVA groups (**Figure 5D**). These data suggest that vitamin D may have exerted a stronger influence on small-airway function in our OVA + ozone mouse model.

### 3.7 The effects of vitamin D on airway inflammation in mouse lung tissue

**Figure 6:**
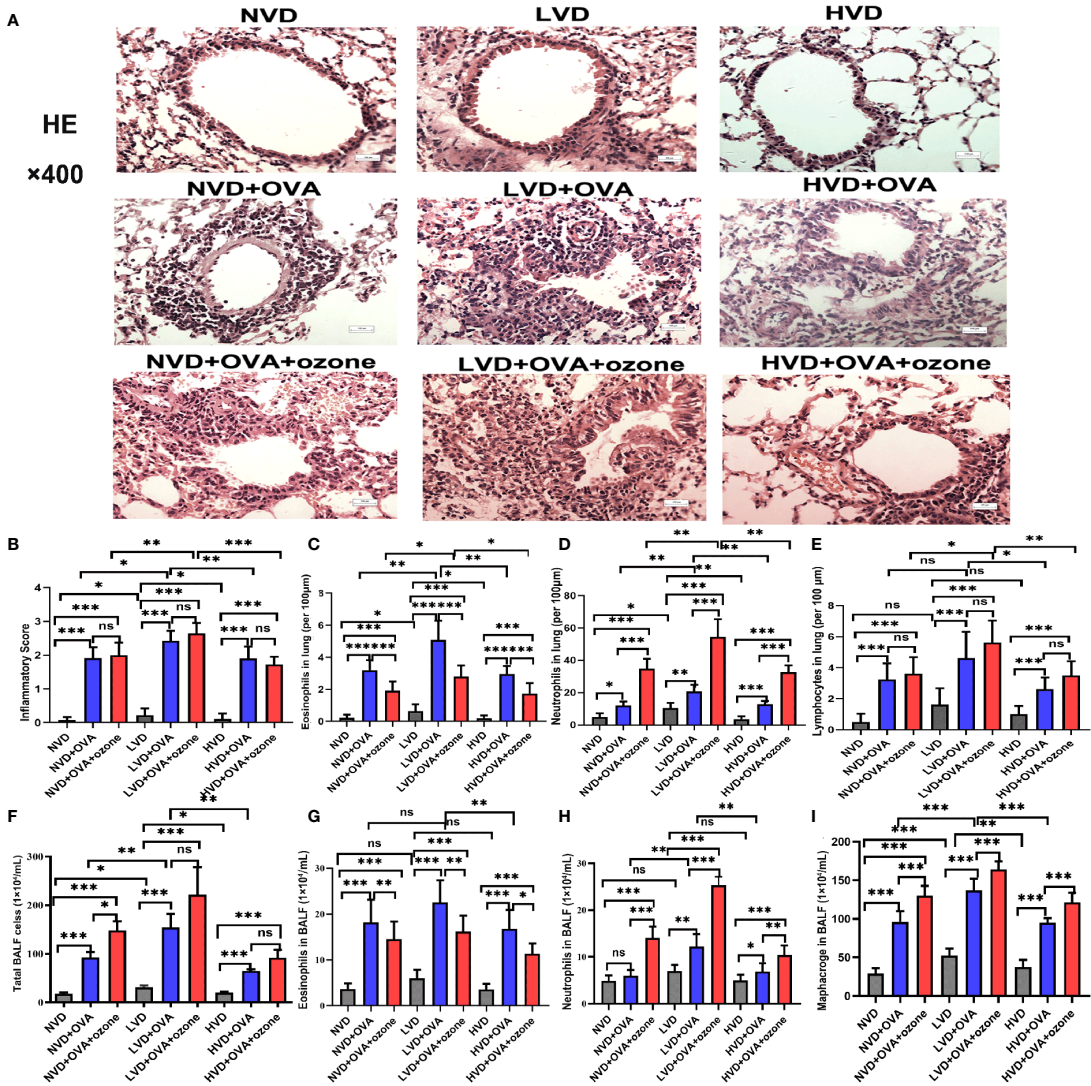
Effects of different levels of vitamin D in the diets on inflammatory cell infiltration in the lung tissue and BALF in T2-high and T2-low asthma models. Airway inflammation in the lung tissue was measured through H&E staining and compared between groups. Airway inflammatory cell infiltration in the BALF was evaluated by Wright–Giemsa staining and compared between groups. **(A)** Representative photomicrographs of HE-stained inflammatory cell infiltration into lung tissue. **(B)** Airway inflammation scores in lung tissue. **(C)** Infiltration density of eosinophils in lung tissue. **(D)** Infiltration density of neutrophils in lung tissue. **(E)** Infiltration density of lymphocytes in lung tissue. **(F)** The number of total cells in BALF. **(G)** Eosinophils in BALF. **(H)** Neutrophils in BALF. **(I)** Macrophages in BALF. Airway inflammatory scores in **(B)** were written as medians as they did not fit a Gaussian distribution; other data are written as mean ± SD. *P < 0.05, **P < 0.01, ***P < 0.001. Scale bar = 100 µm. ns, non-significant. BALF, bronchoalveolar lavage fluid; HE, hematoxylin and eosin.

The LVD control group exhibited more airway inflammation than the other two control groups (**Figure 6A**). The mice fed LVD diets exhibited greater airway inflammation, as represented by increased infiltration of inflammatory cells (e.g., eosinophils and neutrophils) in the lung tissue, than those with NVD and HVD diets in the OVA and OVA + ozone groups (**Figures 6A-D**). However, the lymphocyte infiltration measured in the LVD + OVA group and NVD + OVA group was equivalent (**Figure 6E**). The LVD + OVA group had greater eosinophil infiltration than other groups (**Figure 6C**), but more neutrophils only in the LVD + OVA + ozone group (**Figure 6D**).

### 3.8 The effects of vitamin D on airway inflammation in mouse BALF

There were no significant differences in eosinophils and neutrophils in the BALF among LVD, NVD and HVD control groups. BALF total cell count, eosinophil count and macrophage count were significantly increased after an OVA sensitization/challenge with or without ozone exposure (**Figures 6F, G, I**), but except the number of neutrophils in the NVD + OVA group (**Figure 6H**). However, the BALF total cell count were no obvious differences between OVA group and OVA + ozone group in the LVD and HVD groups. In both asthmatic models, mice fed LVD diets had higher total cell counts, eosinophil counts, neutrophils counts and macrophage counts than mice in the HVD groups (**Figures 6F–I**), but eosinophil counts were equivalent in the LVD and NVD groups (**Figure 6G**). Neutrophil counts were significantly higher in the LVD + OVA + ozone group compared with all other groups (**Figure 6H**), and the eosinophil counts were higher in the LVD + OVA group (**Figure 6G**). Thus, the effects of Vitamin D on airway inflammation measured using BALF were basically the same as its impact on lung tissue.

### 3.9 The effects of vitamin D on inflammatory cytokines levels in mouse BALF

**Figure 7:**
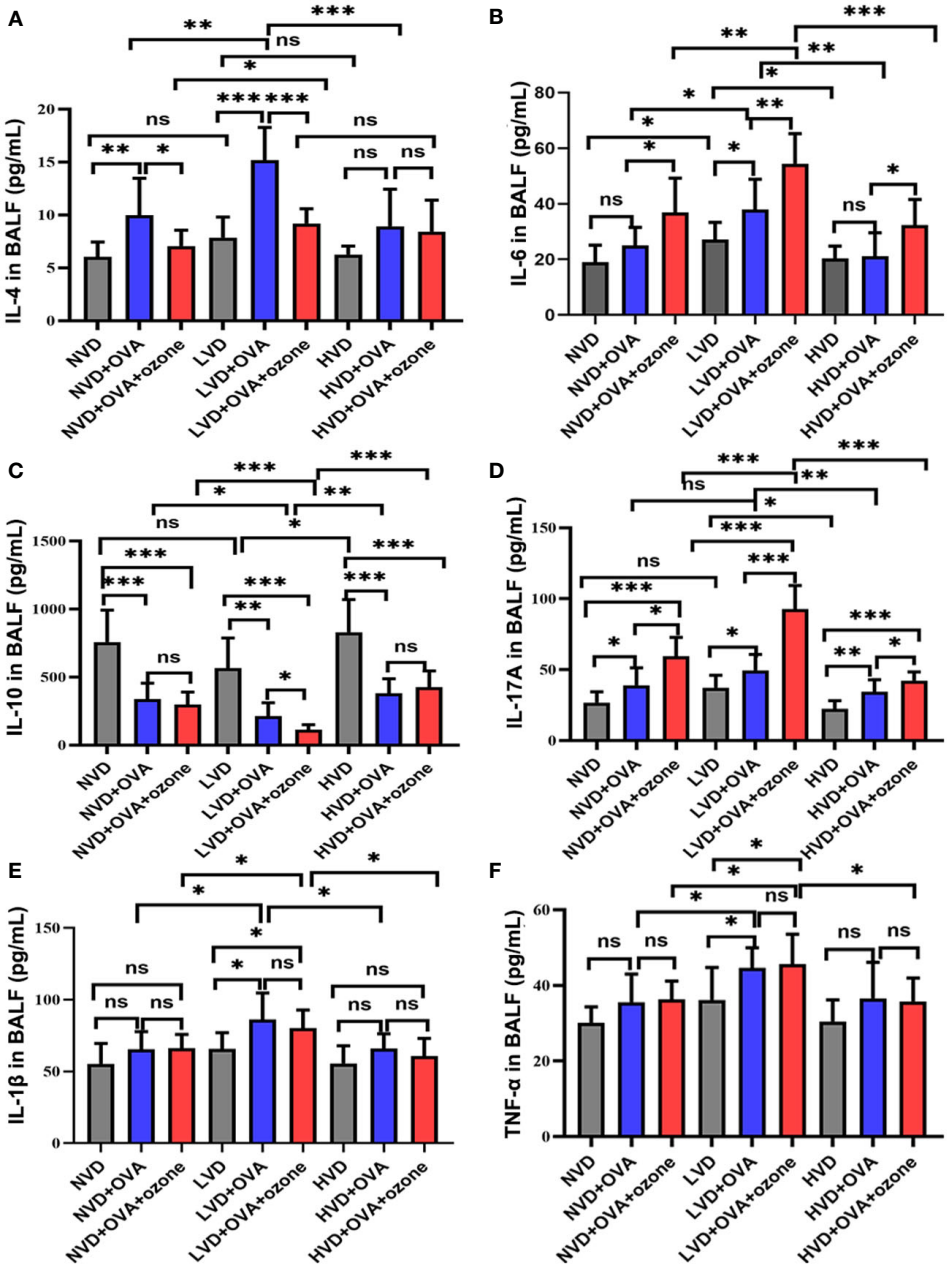
Effects of different levels of vitamin D in the diets on inflammatory cytokine levels in BALF in T2-high and T2-low asthma models. The inflammatory cytokine levels in BALF were analyzed by ELISAs and compared between groups. **(A)** Expression of IL-4 in BALF; **(B)** Expression of IL-6 in BALF; **(C)**Expression of IL-10 in BALF; **(D)** Expression of IL-17A in BALF; **(E)** Expression of IL-1β in BALF; and **(F)** Expression of TNF-α in BALF. Data were presented as mean ± SD. *P < 0.05, **P < 0.01, ***P < 0.001. ns, non-significant. BALF, bronchoalveolar lavage fluid; ELISA, Enzyme-linked immunosorbent assay; IL, interleukin; TNF, Tumor Necrosis Factor.

The expression of the Th2-related cytokine IL-4 in the BALF were no significant differences among LVD, NVD and HVD control groups. However, the expression of IL-4 was significantly elevated in the LVD + OVA group compared with the LVD control group and the LVD + OVA + ozone group (**Figure 7A**). The expression of the Th2-related cytokine IL-6 and the Th17-related cytokine IL-17A were significantly increased in the LVD control groups compared with the HVD control groups, and the expression of IL-6 and IL-17A in the LVD group further increased after OVA or OVA + ozone exposure, in particular in the OVA + ozone group. However, compared with the corresponding control group, the expression of IL-6 in NVD and HVD groups were no significant differences were observed after OVA sensitization/challenge (**Figure 7B**). Meanwhile, there were no significant differences in the expression of IL-17 A between NVD and LVD groups, no obvious differences were also observed after OVA sensitization/challenge (**Figure 7D**). IL-10 (a cytokine linked with T regulatory cells) expression in the BALF of the OVA and OVA + ozone groups was notably lower than controls, and mice fed LVD diets had lower levels of IL-10 than those fed NVD or HVD diets in both asthmatic models, especially the OVA + ozone group. However, the expression of IL-10 was equivalent between the NVD + OVA group and the HVD + OVA group (**Figure 7C**). The expression of the Th1-related cytokines IL-1β and TNF-α were significantly higher in the LVD groups compared with the NVD groups in both asthmatic models, but the expression of TNF-α in the LVD group was significantly higher than the HVD group only among mice exposed to OVA + ozone (**Figures 7E, F**). These data suggest that vitamin D may have an effect on inflammatory cytokine expression in the BALF of Mice exposed to OVA + ozone.

## 4 Discussion

This study is the first to report associations between vitamin D status and airway inflammation, airway resistance (R_L_), and small-airway function measured in asthmatic humans and mouse models with the two common endotypes of asthma. It was observed that vitamin D deficiency in asthmatic patients may induce airway inflammation, small-airway dysfunction, and increased R_L_ in both common asthma endotypes. Further, our animal experiment suggested that different vitamin D diets initiated during lactation and early life impacted airway inflammation and R_L_.

Previous studies (39–41) have shown that low serum vitamin D was associated with worse lung function and severe asthma exacerbations. In our study, patients with lower 25(OH)D levels (Lo) had lower FEV_1_%pred, MMEF%pred, and FEF50%pred compared with those with higher 25(OH)D levels (Hi) in both asthmatic endotypes. However, our results are inconsistent with the discoveries of Castro et al’s (42) that treatment with vitamin D had no significant effect on lung function or airway hyperreactivity, and neither asthma quality of life nor asthma control was improved with vitamin D. IL-17A has been established as an independent risk factor for severe asthma, and has been associated with a T2-low endotype and neutrophilic phenotype (43, 44). In our study, the expression of IL-17A was higher in the T2-low group with Lo compared with Hi, and inversely correlated with serum 25(OH)D level. IL-10 plays a critical role in immunosuppression, and has been shown to regulate cellular sensitivity to glucocorticoids in lymphocytes and monocytes (45). Our study showed that the expression of IL-10 was lower in the T2-low group with Lo than with Hi, a relationship not seen in the T2-high group. This is consistent with Creed TJ et, al (45) suggested that vitamin D might help to alleviate airway inflammation and revert steroid-resistance through elevating the level of IL-10. Therefore, vitamin D may have an important role in T2-low asthma. However, Th2-related cytokine IL-5 were significantly increased in vitamin D-deficient patients in T2-high group, but the effect no significant difference in Th2-related cytokine IL-4, its mechanism needed to further study. Our clinical study found that vitamin D status had a stronger correlation with FEV_1_%pred in the T2-low group. Furthermore, the small-airway functional variable MMEF%pred was positively linked with 25(OH)D status only in the T2-low group. Our results also demonstrated that FEV_1_%pred was negatively correlated with eosinophil count and positively associated with IL-10 in the T2-high group. However, small-airway variables such as MMEF%pred and FEF50%pred did not correlate with airway inflammation severity in this group. In contrast, MMEF%pred was negatively correlated with neutrophil count and IL-17A in the T2-low group. Although FEV_1_%pred was associated with airway inflammation in both asthmatic groups, small-airway dysfunction may permit the evaluation of illness severity or contribute to the progression of airway inflammation only in T2-low asthma. Our studies suggest that vitamin D may have a positive impact on FEV_1_%pred and small-airway dysfunction *via* its inhibitory effect on airway inflammation, especially in patients with the T2-low asthma endotype.

As our clinical data on asthmatic patients was sparse and retrospective, we relied on animal experiments. The mechanisms behind the relationship between vitamin D and asthma require further investigation, particularly with respect to different asthma endotypes. Our pre-clinical model showed that serum 25(OH)D levels in vitamin D deficient (LVD) group were lower than in the vitamin D sufficient (NVD) and vitamin D supplemented (HVD) groups in both asthmatic models. This finding is consistent with those of Kim et al. (29), who showed that serum 25(OH)D concentrations were significantly higher in groups supplemented with vitamin D compared with non-supplemented groups. Further, mice in the LVD group had significantly increased R_L_ and airway inflammation compared with those in the NVD and HVD groups. This is consistent with our clinical findings. Mice fed LVD diets had increased inflammation, including eosinophil, neutrophil and lymphocyte infiltration, in their lung tissues compared with those fed HVD diets. The LVD + OVA + ozone (T2-low) group had a higher inflammation score than the LVD + OVA (T2-high) group. Poon et al. (46) showed that vitamin D has its anti-proliferative and anti-inflammatory effects on airway smooth muscle (ASM) cells in the setting of inflammation and airway remodeling. This study suggests that vitamin D deficiency may increase airway inflammation, especially after OVA + ozone exposure. Meanwhile, the LVD + OVA + ozone group had an increased total, neutrophil, and macrophage counts and fewer eosinophils in their BALF than the LVD + OVA group. This correlates with findings by Brehm et al. (47), who showed that vitamin D status was negatively related to peripheral blood neutrophil or eosinophil counts in asthmatic children. These results suggest that vitamin D deficiency might influence asthma pathogenesis by modulating crucial processes that influence airway inflammation. The mice fed LVD diets had also increased R_L_ and decreased -logPC100 compared with those fed HVD diets after OVA alone or OVA + ozone, suggesting that the Ach responsiveness induced by OVA alone or OVA + ozone could be enhanced by LVD. Our results are in agreement with a previous study that showed that a continuous vitamin D deficiency increased the development of AHR in asthma models (19, 48). Mice fed LVD diets also reduced small-airway function than those fed HVD diets in both asthma models. The HVD + OVA + ozone group had a lower MMEF than the HVD + OVA group, and the LVD + OVA + ozone group had a lower FEF50 than the LVD control group, but this relationship was not seen in the LVD+OVA group. This study therefore suggests that vitamin D deficiency affects the small-airway function asthmatics, especially with the T2-low endotype. The mice fed LVD diets after OVA alone or OVA + ozone had increased IL-4 expression compared with those fed HVD diets. Further, IL-10 expression in both asthmatic models fed LVD diets was lower than those fed HVD diets. This is consistent with previous studies (49) suggested that vitamin D may help reduce airway inflammation and reverse steroid-resistance by increasing IL-10 expression. IL-6 and IL-17A expression were increased in the LVD + OVA + ozone group compared with the LVD + OVA group, suggesting that LVD + OVA + ozone seems to induce a greater inflammatory response than LVD + OVA. In our animal experiment, vitamin D also affect airway inflammation and R_L_ on T2-high asthma, however, vitamin D deficiency may therefore play a more important role in the pathogenesis of T2-low asthma.

Current guidelines disagree regard the optimal dosage and concentration of 25(OH)D (50). Vitamin D at 1000 IU/kg appeared to effectively improve the effects of vitamin D deficiency in our mouse model. This supports findings that high dose vitamin D supplementation (administered at a dose of 2280 IU/kg) (31) was required to prevent the development of asthma in offspring than vitamin D sufficiency alone. This may support the clinical rationale for providing effective vitamin D supplementation to prevent infant asthma. It can also be inferred that higher concentrations of vitamin D may be necessary to modulate the airway inflammation and resistance in mouse models, especially those replicating T2-low asthma. Vitamin D has a well-documented role in the regulation of the adaptive T-cell response and innate immunity. However, no specific function has been identified for why vitamin D levels impact Th2-mediated eosinophilic and Th17-mediated neutrophilic airway inflammation. Fawaz and colleagues (18) identified a potential protective role of vitamin D through the modulation of the pathogenic T cell response. Our results suggest that vitamin D deficiency is associated with increased R_L_, the pro-inflammatory Th2 and Th17 responses, and the regulation of the T-reg production of IL-10 in clinical and pre-clinical models. Our study also showed that airway obstruction was related to different airway inflammation cytokines in both asthmatic endotypes. Vitamin D may therefore improve airway obstruction *via* regulation of different airway inflammatory pathways in both asthmatic endotypes, but it may exert a greater impact on T2-low asthma, although its mechanism of action requires further research.

There are several limitations to our study. First, few studies have evaluated if vitamin D supplementation can improve the airway inflammation and R_L_ of mouse models with vitamin D deficiency. Secondly, further studies are required to determine which asthma endotype would benefit the most from vitamin D supplementation. Lastly, we only performed an observational study on the association between vitamin D deficiency and both asthmatics endotypes. The potential association between vitamin D and T2-low asthma requires further study.

## 5 Conclusion

Vitamin D-deficiency induced inflammatory cytokine expression, and correlated with airway resistance in both asthma endotypes. The potential effects of vitamin D on airway obstruction may be through changes in inflammatory cytokines and is more evident in T2-low asthma. As there are few treatment options remain for T2-low asthma. The mechanisms behind the interactions between vitamin D and both asthma endotypes should be studied individually, and further studies on the potential signaling pathways involved in vitamin D and T2-low asthma are warranted.

## Data availability statement

The original contributions presented in the study are included in the article/**Supplementary Material**. Further inquiries can be directed to the corresponding author.

## Ethics statement

The studies involving human participants were reviewed and approved by the Institutional Review Board at Shanghai General Hospital (No. 2018KY186) and registered on chictr.org.cn (No. ChiCTR2000029065). Written informed consent to participate in this study was provided by the participants’ legal guardian/next of kin.The animal study was reviewed and approved by Committee for Animal Studies at Shanghai General Hospital, China. Written informed consent was obtained from the owners for the participation of their animals in this study.

## Author contributions

YZ, YQ, WB and MZ conceived of and designed the entire study. YYZ, XT, CL and DY contributed to data collection and statistical analyses. YX, YQ, LH, and QF performed the experiments. YZ and YQ wrote the manuscript, supervised by MZ. All authors agreed to be responsible for all aspects of the work in ensuring that questions related to the integrity or accuracy of any part of the work are appropriately investigated and resolved. All authors contributed to the article and approved the submitted version.
