# Supplementary material for: Evaluating the effects of vitamin D Level on airway obstruction in two asthma endotypes in humans and in two mouse models with different intake of vitamin D during early-life
Source: Front Immunol. 2023 Jan 30;14:1107031. doi: 10.3389/fimmu.2023.1107031 (PMC9922677; doi:10.3389/fimmu.2023.1107031)
Supplement: Supplementary file 2 [file DataSheet_2.docx]

**
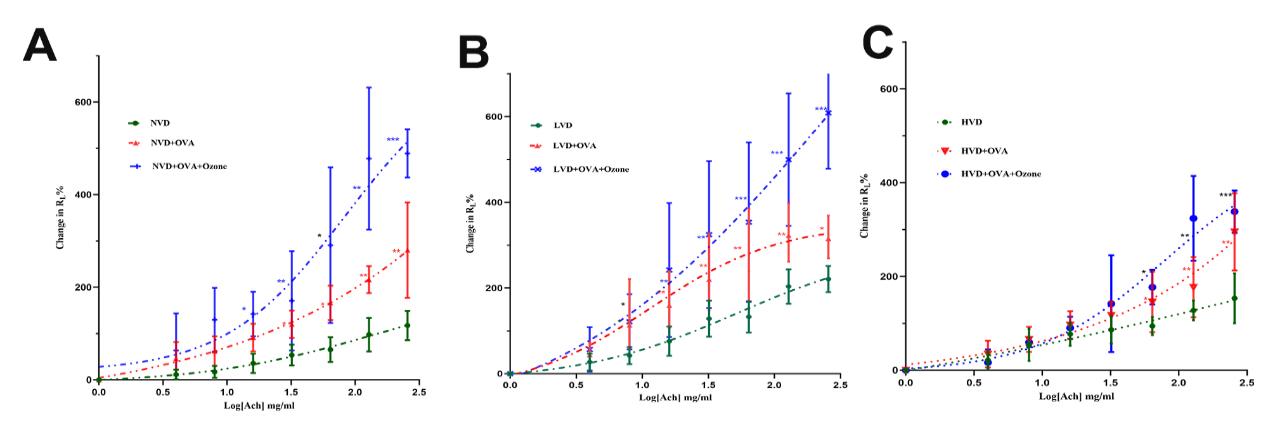
**

**Figure S2：**Effects of different vitamin D diets on R_L_. Mean percentage increase in R_L_ to inhaled ACh as the concentration gradually increased (4, 8, 16, 32, 64, 128 and 256 mg/mL). (A) Effects of NVD diets on R_L_; (B) Effects of LVD diets on R_L_; (A) Effects of HVD diets on R_L_.
