# Supplementary material for: Evaluating the effects of vitamin D Level on airway obstruction in two asthma endotypes in humans and in two mouse models with different intake of vitamin D during early-life
Source: Front Immunol. 2023 Jan 30;14:1107031. doi: 10.3389/fimmu.2023.1107031 (PMC9922677; doi:10.3389/fimmu.2023.1107031)
Supplement: Supplementary file 3 [file Table_1.docx]

***Table S1.*** ***Composition of the experimental diets****

| Ingredient | Vitamin D sufficient diet (NVD) | Vitamin D deficient diet (LVD) | Vitamin D supplemented diet (HVD) |
| --- | --- | --- | --- |
| Vitmain A, Acetate (500,000 IU/g) | 4000 IU Vit A | 4000 IU Vit A | 4000 IU Vit A |
| Vitamin D3 (100,000 IU/g) | 1000 IU Vit D3 | 0 IU Vit D3 | 2280 IU Vit D3 |
| Vitamin E Acetate (500 IU/g) | 75 IU Vit E | 75 IU Vit E | 75 IU Vit E |
| Phylloquinone | 0.75 mg | 0.75 mg | 0.75 mg |
| Biotin,1.0% | 0.2 mg Biotin | 0.2 mg Biotin | 0.2 mg Biotin |
| Cyanocobalamin,0.1% | 25 μg Vit B12 | 25 μg Vit B12 | 25 μg Vit B12 |
| Folic Acid | 2 mg Folic Acid | 2 mg Folic Acid | 2 mg Folic Acid |
| Nicotinic Acid | 30 mg Nicotinic Acid | 30 mg Nicotinic Acid | 30 mg Nicotinic Acid |
| Calcium Pantothenate | 16 mg Pantothenic Acid | 16 mg Pantothenic Acid | 16 mg Pantothenic Acid |
| Pyridoxine-HCI | 7 mg Vit B6 | 7 mg Vit B6 | 7 mg Vit B6 |
| Riboflavin | 6 mg Vit B2 | 6 mg Vit B2 | 6 mg Vit B2 |
| Thiaminn HCI | 6 mg Vit B1 | 6 mg Vit B1 | 6 mg Vit B1 |

***Resource: Research Diets Inc., New Brunswick, NJ, USA**

***Use at 10 gm/kg diet or 10 gm/ 4000 kcal digestible energy.**
