# Supplementary material for: Evaluating the effects of vitamin D Level on airway obstruction in two asthma endotypes in humans and in two mouse models with different intake of vitamin D during early-life
Source: Front Immunol. 2023 Jan 30;14:1107031. doi: 10.3389/fimmu.2023.1107031 (PMC9922677; doi:10.3389/fimmu.2023.1107031)
Supplement: Supplementary file 4 [file Table_2.docx]

**Table S2. Sex ration of mice**

|  |  | 8 weeks |  |  |  | 12 weeks |  |
| --- | --- | --- | --- | --- | --- | --- | --- |
|  | Total | Male | Female |  | Total | Male | Female |
| NVD (g) | 22.56±3.25 (n=10) | 25.16±2.29 (n=5) | 20.27±1.63 (n=5) |  | 26.51±3.35 (n=8) | 29.22±1.13 (n=4) | 22.98±1.78 (n=4) |
| LVD (g) | 21.62±3.04 (n=10) | 24.26±2.34 (n=5) | 19.97±1.45 (n=5) |  | 26.63±3.71 (n=8) | 30.20±1.54 (n=4) | 22.09±2.27 (n=4) |
| HVD (g) | 23.22±3.22 (n=10) | 24.67±2.78 (n=5) | 20.66±1.77  (n=5) |  | 27.13±3.22 (n=8) | 29.18±2.11 (n=4) | 23.12±2.25 (n=4) |
| NVD+OVA (g) | 22.58±3.34 (n=10) | 25.13±2.16 (n=5) | 19.99±2.01 (n=5) |  | 27.11±3.76 (n=8) | 29.57±2.47 (n=4) | 23.44±2.00 (n=4) |
| LVD+OVA (g) | 22.04±3.13 (n=10) | 25.18±2.37 (n=5) | 19.90±1.37 (n=5) |  | 25.94±4.18 (n=8) | 28.59±3.21 (n=4) | 22.67±2.66 (n=4) |
| HVD+OVA (g) | 22.59±3.50 (n=10) | 25.08±2.84 (n=5) | 20.33±2.11 (n=5) |  | 27.07±2.69 (n=8) | 28.74±2.22 (n=4) | 23.53±1.87 (n=4) |
| NVD+OVA+ozone (g) | 22.55±3.22 (n=10) | 25.37±2.74 (n=5) | 20.43±2.08 (n=5) |  | 27.13±2.88 (n=8) | 29.12±2.09 (n=4) | 23.18±1.99 (n=4) |
| LVD+OVA+ozone (g) | 21.98±3.55 (n=10) | 25.09±2.86 (n=5) | 19.94±1.91 (n=5) |  | 26.47±2.35 (n=8) | 28.88±2.31 (n=4) | 22.68±2.33 (n=4) |
| HVD+OVA+ozone (g) | 23.05±3.18 (n=10) | 26.08±2.37 (n=5) | 20.22±2.07 (n=5) |  | 27.33±2.56 (n=8) | 30.52±2.38 (n=4) | 23.49±2.45 (n=4) |
| **Data were expressed as mean ± SD** | | |  |  |  |  |  |
| **There is no statistical difference in body weight among the six groups at 8 weeks old or 12 weeks old (*P* > 0.05)**  **There was no significant difference in the proportion of male and female offspring among the groups (*P* > 0.05)** | | | | | | | |
