# Supplementary material for: Evaluating the effects of vitamin D Level on airway obstruction in two asthma endotypes in humans and in two mouse models with different intake of vitamin D during early-life
Source: Front Immunol. 2023 Jan 30;14:1107031. doi: 10.3389/fimmu.2023.1107031 (PMC9922677; doi:10.3389/fimmu.2023.1107031)
Supplement: Supplementary file 5 [file Table_3.docx]

| **Table S3- P value of correlation analysis in Figure 3A** |
| --- |

|  | **25(OH)D** | **Eosinophils** | **Neutrophils** | **FeNO** | **FEV_1_%**  **pred** | **MMEF%pred** | **FEF50%pred** | **IL-4** | **IL-5** | **IL-1β** | **IL-6 IL-10 IL-17A TNF-α** |
| --- | --- | --- | --- | --- | --- | --- | --- | --- | --- | --- | --- |
| **25(OH)D** | **0** |  |  |  |  |  |  |  |  |  |  |
| **Eosinophils** | **0.017** |  |  |  |  |  |  |  |  |  |  |
| **Neutrophils** | **0.126** | **0.133** |  |  |  |  |  |  |  |  |  |
| **FeNO** | **0.693** | **0.043** | **0.621** |  |  |  |  |  |  |  |  |
| **FEV_1_%pred** | **0.001** | **0.001** | **0.403** | **0.440** |  |  |  |  |  |  |  |
| **MMEF%pred** | **0.989** | **0.617** | **0.988** | **0.134** | **0.676** |  |  |  |  |  |  |
| **FEF50%pred** | **0.619** | **0.788** | **0.640** | **0.112** | **0.831** | **0.000** |  |  |  |  |  |
| **IL-4** | **0.912** | **0.030** | **0.177** | **0.917** | **0.602** | **0.371** | **0.532** |  |  |  |  |
| **IL-5** | **0.016** | **0.004** | **0.384** | **0.751** | **0.130** | **0.635** | **0.497** | **0.030** |  |  |  |
| **IL-1β** | **0.474** | **0.205** | **0.139** | **0.793** | **0.182** | **0.942** | **0.752** | **0.167** | **0.011** |  |  |
| **IL-6** | **0.802** | **0.238** | **0.360** | **0.847** | **0.169** | **0.749** | **0.753** | **0.901** | **0.748** | **0.212** |  |
| **IL-10** | **0.017** | **0.140** | **0.841** | **0.032** | **0.006** | **0.345** | **0.302** | **0.793** | **0.202** | **0.187** | **0.770** |
| **IL-17A** | **0.452** | **0.522** | **0.811** | **0.803** | **0.198** | **0.313** | **0.226** | **0.177** | **0.306** | **0.473** | **0.514 0.618** |
| **TNF-α** | **0.001** | **0.013** | **0.623** | **0.059** | **0.296** | **0.943** | **0.907** | **0.675** | **0.104** | **0.515** | **0.229 0.441 0.185 0** |
