# Supplementary material for: Evaluating the effects of vitamin D Level on airway obstruction in two asthma endotypes in humans and in two mouse models with different intake of vitamin D during early-life
Source: Front Immunol. 2023 Jan 30;14:1107031. doi: 10.3389/fimmu.2023.1107031 (PMC9922677; doi:10.3389/fimmu.2023.1107031)
Supplement: Supplementary file 6 [file Table_4.docx]

**Table S4- P value of correlation analysis in Figure 3B**

|  | **25(OH)D** | **Eosinophils** | **Neutrophils** | **FeNO** | **FEV_1_%**  **pred** | **MMEF%pred** | **FEF50%pred** | **IL-4** | **IL-5** | **IL-1β** | **IL-6 IL-10 IL-17A TNF-α** |
| --- | --- | --- | --- | --- | --- | --- | --- | --- | --- | --- | --- |
| **25(OH)D** | **0** |  |  |  |  |  |  |  |  |  |  |
| **Eosinophils** | **0.598** |  |  |  |  |  |  |  |  |  |  |
| **Neutrophils** | **0.001** | **0. 336** |  |  |  |  |  |  |  |  |  |
| **FeNO** | **0.405** | **0.029** | **0.185** |  |  |  |  |  |  |  |  |
| **FEV_1_%pred** | **0.033** | **0.453** | **0.618** | **0.963** |  |  |  |  |  |  |  |
| **MMEF%pred** | **0.022** | **0.246** | **0.040** | **0.690** | **0.636** |  |  |  |  |  |  |
| **FEF50%pred** | **0.109** | **0.364** | **0.288** | **0.873** | **0.928** | **0.000** |  |  |  |  |  |
| **IL-4** | **0.334** | **0.338** | **0.726** | **0.073** | **0.110** | **0.631** | **0.808** |  |  |  |  |
| **IL-5** | **0.232** | **0.232** | **0.426** | **0.055** | **0.254** | **0.126** | **0.623** | **0.019** |  |  |  |
| **IL-1β** | **0.800** | **0.352** | **0.890** | **0.996** | **0.030** | **0.636** | **0.968** | **0.406** | **0.103** |  |  |
| **IL-6** | **0.169** | **0.383** | **0.835** | **0.913** | **0.623** | **0.303** | **0.199** | **0.796** | **0.035** | **0.108** |  |
| **IL-10** | **0.008** | **0.914** | **0.003** | **0.518** | **0.008** | **0.558** | **0.837** | **0.425** | **0.537** | **0.284** | **0.732** |
| **IL-17A** | **0.040** | **0.199** | **0.026** | **0.217** | **0.047** | **0.026** | **0.210** | **0.526** | **0.002** | **0.061** | **0.007 0.260** |
| **TNF-α** | **0.259** | **0.397** | **0.853** | **0.393** | **0.076** | **0.182** | **0.109** | **0.067** | **0.006** | **0.196** | **0.087 0.696 0.110 0** |
